# The air we breathe: understanding the impact of the environment on pneumonia

**DOI:** 10.1186/s41479-022-00094-1

**Published:** 2022-02-01

**Authors:** Lidwien A. M. Smit

**Affiliations:** Institute for Risk Assessment Sciences, Utrecht University, Utrecht, The Netherlands

**Keywords:** Air pollution, Epidemiology, Farming

## Abstract

An increased risk of community-acquired pneumonia has been shown in residents of rural livestock farming areas in the Netherlands and United States, probably due to air pollution exposure or zoonotic infections. Spatial epidemiological analyses have particularly implicated poultry and goat farms in the increased risk—an observation that warrants further research. Studying the viral or bacterial etiology of community-acquired pneumonia using traditional microbiological methods or metagenomic sequencing could help to fathom to what extent environmental factors and causative pathogens contribute to spatial differences in the incidence of severe acute respiratory infections.

## Main text

When the World Health Organization (WHO) presented the new Global Air Quality Guidelines in September 2021, they emphasized “the clear evidence of the damage air pollution inflicts on human health, at even lower concentrations than previously understood” [1]. Although associations with lower respiratory infection risk are generally small, outdoor and indoor air pollution are ubiquitous and therefore contribute to a substantial burden of pneumonia and bronchiolitis worldwide [2]. Agriculture is a major—but often overlooked—source of air pollution due to atmospheric emissions of coarse and fine particulate matter and reactive nitrogen [3, 4]. An increased risk of pneumonia in residents of rural livestock farming areas has been shown in several studies in the Netherlands and United States [5–8], probably due to air pollution exposure or zoonotic infections, although there is limited evidence to support a zoonotic etiology apart from outbreak situations [9]. Spatial epidemiological analyses have particularly implicated poultry and goat farms in the increased risk of pneumonia, an observation that warrants further research.

In a recent article in *Pneumonia* [10] Roof et al. have attempted to shed more light on the viral or bacterial etiology of hospitalized community-acquired pneumonia (CAP) patients living in a livestock farming area in the Netherlands. Analysis of routine laboratory results of two hospitals did not reveal clear differences in identified micro-organisms between patients living within two kilometers of goat or poultry farms and patients living further away from those farms. The percentage of positive urine antigen tests for *Streptococcus pneumoniae* was slightly higher in patients living close to goat farms (15.2% vs. 11.3%; *p* = 0.10) and poultry farms (14.4% vs. 11.3%; *p* = 0.14). When laboratory data from the Jeroen Bosch Hospital—the hospital with the higher livestock farm density in its catchment area—alone were analyzed, a larger percentage of positive pneumococcal urine antigen tests in patients living close to poultry farms was found (15.6% vs. 10.1%; *p* = 0.03).

Identification of pathogens was carried out in accordance with normal clinical practice, and not as part of a systematic research initiative aiming to detect causative micro-organisms in a large proportion of CAP patients. While convenient, the use of routine diagnostic data for CAP research has shortcomings. The low pathogen detection rate, 25%, is consistent with previous studies on ‘real-life’ testing practices in adults hospitalized with CAP [11], but test results available from both hospitals participating in this retrospective study only allowed spatial comparisons for *S. pneumoniae* and *Legionella pneumophila* (urinary antigen tests), and Influenza A and B (PCR). A nationwide severe acute respiratory infections surveillance system—including a broad range of viral, bacterial, and fungal test results—is currently not available, but would be of great value for preparedness and emergency response, and for early detection of signals that require further investigation [12]. Such surveillance data could also be advantageous for studying various environmental risk factors of pneumonia. For example, use of antibiotics possibly prescribed for pneumonia (amoxicillin, doxycycline, and co-amoxiclav) was 5–10% higher among people living close to poultry farms in a nationwide Dutch study [13]. However, the presence of poultry farms explained only a limited amount of the total variation in antibiotic use, while remarkable regional differences were observed with high use patterns in areas with a generally low socio-economic status. Availability of detailed surveillance data could help to fathom to what extent environmental factors and causative pathogens contribute to spatial and temporal differences in the incidence of severe acute respiratory infections.

Despite the abovementioned limitations in data quality, the slightly higher percentage of positive urine antigen tests for *S. pneumoniae* in CAP patients living near poultry [10] is supportive of a previous Dutch hospital-based study that used 16S rRNA gene sequencing to analyze the oropharyngeal microbiota composition of 126 CAP patients [5]. At the time of hospital admission and before taking antibiotics, a higher relative abundance of *S. pneumoniae* and overrepresentation of a microbiota composition cluster dominated by *S. pneumoniae* was found in CAP patients living near a poultry farm [5]. Complementary to the traditional microbiological methods used in the study by Roof et al. [10], next generation sequencing has emerged as a powerful tool to study whether imbalance of microbial communities is predictive of respiratory infections. In line with animal and in vitro experiments, the increased abundance of *S. pneumoniae* in CAP patients living close to a poultry farm suggests that farm emissions such as particulate matter and endotoxin may be involved in alterations of the upper respiratory tract microbiota composition, possibly leading to increased risk of infection [5]. In future research, metagenomic sequencing methods also have the potential to identify pathogens in the large proportion of CAP patients with unknown etiology, which may reveal new leads to understanding how the environment contributes to pneumonia development.

While researchers must continue to investigate the impact of the living environment on pneumonia risk, the revised WHO Air Quality Guidelines underscore that action is needed to mitigate adverse health effects of air pollution. In response to public health concerns, the Dutch government has announced ambitions to reduce the high particulate matter emissions from poultry farms [3]. The emission reduction targets have already stimulated initiatives in the sector to develop poultry dust reduction techniques which if effective, should ultimately lead to improved air quality in livestock farming areas.

## Data Availability

Not applicable

## Abbreviations

CAP: Community-acquired pneumonia
WHO: World Health Organization
